# Challenges associated with the implementation of occupational safety and health management systems in manufacturing industry of Mutare, Zimbabwe

**DOI:** 10.3389/fpubh.2025.1587769

**Published:** 2025-07-04

**Authors:** Johanes Mandowa, Mark Matsa, Steven Jerie

**Affiliations:** Department of Geography, Environmental Sustainability and Resilience Building, Midlands State University, Gweru, Zimbabwe

**Keywords:** occupational safety and health, occupational safety and health management system, occupational safety and health management systems, preventive safety culture, problem driven iterative adaptation (PDIA), OSH practitioner, Zimbabwe

## Abstract

**Objective:**

This study examines challenges of implementing the Occupational Safety and Health Management System (OSHMS) in the manufacturing industry of Mutare, Zimbabwe as well as to develop a framework to circumvent the identified challenges.

**Methods:**

Utilizing a descriptive mixed-method design, data was collected through questionnaires, interviews, and observations, alongside secondary sources like company OSH records. Primary and secondary data was analyzed and discussed continuously in a descriptive prose. Chi-square test at 5% significance level was applied to test inferential statistics and the test results were described qualitatively to compliment other data sources.

**Results:**

Key findings identified inadequate resources, lack of leadership and commitment, insufficient employee involvement, and a shortage of qualified OSH practitioners as major obstacles. The study reveals that causes of these challenges are multifactorial, necessitating comprehensive interventions. A lack of a preventive safety culture emerged as a critical underlying factor affecting OSHMSs implementation.

**Conclusion:**

In alignment with Article 14 of ILO Convention 155, the research advocates for a national strategy to foster a preventive OSH culture, integrating OSH into all education and training levels. Additionally, it recommends that the Government of Zimbabwe ratify ILO Convention 187 to support the establishment of a national OSH qualifications framework, enhancing OSH management at both national and workplace levels. This study originality is its ability to develop a problem driven framework of interventions ideal for solving OSH implementation challenges and their causal factors in the manufacturing industry of Mutare through experiential learning, coordination, and a collaborative approach. The study is of global significance as it has an impact of providing opportunities for sustainable OSH transformation in the manufacturing industry of Mutare and beyond.

## 1 Introduction

The global Occupational Safety and Health (OSH) landscape is becoming more vulnerable owing to the negative effects of emerging complex, dynamic and unprecedented OSH risks (1). In the current work dispensation, the application of OSH management systems (OSHMSs) has been embraced as a panacea to ensure OSH and business sustainability (2). Occupational Safety and Health Management System (OSHMS) is a set of interrelated elements to establish OSH policy, objectives, and programs for the achievement of the policy and objectives. OSH management systems are based on the Plan, Do, Check and Act (PDCA) cycle embodying elements of policy, organizing, planning and implementation, performance measurement, monitoring and reviewing.

Shabani et al. (3) study ventilated the importance of implementing safety and health management programs in bringing about a positive change on employee productivity and organizational performance. Several studies (4–6) have demonstrated the importance of implementing OSHMSs in reducing the incidence of occupational accidents at work. A study by Esterhuyzen and Visser (7), challenged governments, policymakers, practitioners and management teams to consider implementation of occupational safety and health management systems aimed at ensuring improvement in occupational safety and health compliance. Despite this realization on the importance of OSHMSs, low uptake of OSH management systems is still a phenomenon confronting most developing countries (1, 2, 8). Moyo et al. (9) asserted suboptimal to nonexistent level of OSHMSs implementation at many workplaces in SADC member states that include South Africa, Zimbabwe, Botswana, and Zambia. Earlier studies have however shown that implementing an occupational safety and health management system is not easy due to many challenges arising during its implementation (10, 11).

Many studies (4, 6, 10, 12–14) have been conducted globally to explore the challenges associated with implementation of OSHMSs in various industrial sectors. Baghdadi (4) study acknowledged the existence of numerous challenges impeding the successful adoption of OSH measures in workplace environments. Alaloul et al. (13) highlighted environmental challenges such as exposure to extreme weather conditions, high radiation levels and toxic elements as dynamic threats to effective implementation of OSHMSs. Balkhyour et al. (15) and Baghdadi (4) identified scarcity of financial resources, limited availability of material resources and political stability as limitations to OSHMSs implementation in regions marked by conflict or economic development challenges, such as Palestine. A study by Abdi and Hareru (6) highlighted unavailability of personal protective equipment, lack of local guidelines on the standard procedures for OSH, limited expertise, and insufficient historical data availability as the main challenges on the implementation of OSH measures at building construction projects in Somaliland. Different OSH implementation challenges, such as poor safety awareness among leaders, inadequate worker training and ineffective safety regulation implementation were identified by Kheni and Afatsawu (14), and Kunodzia et al. (10) as confronting workplaces in the construction sector.

Safety culture and management commitment has been examined in many contexts that include health care (16), transport (rail traffic, road traffic) (17), the aviation industry (18), the food industry, education, and household accidents (19) as major challenges that influence OSHMSs implementation. Unavailability of good safety culture and management commitment have been identified in several earlier studies (1, 20–25) as evidence for non-existence of an OSHMS. Bautista-Bernal et al. (20) asserted the necessity of developing a safety culture in the company in meeting the needs of employees, and thus improving safety performance. Sugiono et al. (26) posited the positive influence of safety culture in driving effective implementation of OSHMSs as it reflects the attitudes and behaviors of employees toward safety. As noted by Perez (27) non-implementation of OSHMSsis generally evident at workplaces that lack good safety culture. There is no consensus in literature about the concept of safety culture and its measurement (28) owing to its multidimensional character, however there is some convergence of thoughts among many researchers (22, 29–34) on the principal factors that can be used to gauge safety culture at a particular workplace namely; safety rules, reporting unsafe acts and conditions, reporting accidents and incidents, conducting Hazard Identification Risk Assessment (HIRA), employee involvement, peer feedback, safety training, safety communication, utilization of PPE, continuous improvement, safety induction, productivity pressure, and accident investigation.

Management commitment is important in aiding the implementation of OSHMSs at workplaces (1, 8, 35–37). According to Tan et al. (38), management commitment is expressed in a number of ways such as safety education and training, giving safety and health performance rewards and ensuring that employees are empowered by involving them in all facets of OSH management decision making. However, these measures come at considerable cost to the organization and therefore as noted by Abdi and Hareru (6), availability of an OSH budget to unlock the required financial resources to drive the effective implementation of an OSH management system is an indicator of the availability of management commitment at a workplace. AL-Qaisi (39) confirmed the observation by Abdi and Hareru (6) by asserting that provision of necessary resources was an indicator of senior management commitment. On the other hand, management commitment according to Nelson and Zega (40) can also be demonstrated through establishing punitive measures against employees who disobey safety measures such as embarking on at risk behaviors. Literature shows that there is no generally established template to measure management commitment (1). Authoritative sources (35, 36), however presume that management commitment to OSH management can sufficiently be evaluated by considering the following 14 parameters namely Formulation of the SHE policy, visible felt leadership walkabouts, certification of the OSH management system, management participation in safety training, Resources for OSH management (Budget item), Setting of OSH objectives and targets, appointment of an OSH practitioner, OSH appraisal system, Participation in risk assessment, Participation in Safety and Health committee business, Awards/Incentive System and Safety always being regarded as the first item on the agenda in all operational meetings, Management participation in safety inspections and audits and HR policies should embrace OSH (induction, on job safety training, formal training, pre, periodic and exit medicals).

It is critical to note that as observed by Mandowa et al. (1) study, there is limited research that has been conducted to explore in depth the contextual challenges and opportunities to implementation of OSHMSs in least and developing countries. The majority of the studies that attempted to explore OSHMSs implementation challenges were focused mainly on developed countries whose political, social and economic environmental context is diametrically different to that of the least and developing countries. Kunodzia et al. (10) observed that generally, studies concerning challenges to OHSMSs implementation were limited to systematic literature reviews and were conducted in developed countries. This lack of focused research toward in depth understanding OSHMSs implementation challenges in least and developed countries could be the reason that is increasingly contributing to an unsustainable scenario where workplaces in the least and developing countries resort to implementation of off the shelf safety approaches and systems adopted from developed countries that may not be congruent with their political, social and economic environmental context.

Moyo et al. (8) highlighted a plethora of occupational safety and health challenges facing SADC member states that include Occupational Health Services (OHS) human resource capital deficits, lack of comprehensive national and institutional OSH management systems and the proliferation of an unregulated informal economy among others. Shabani et al. (3) study identified challenges faced by organizations in Zimbabwe in implementing OSH programs as lack of resources, inadequate training and lack of awareness among employees about the importance of OSH. Although ILO and its stakeholders recognize the need to ensure OSH sustainability as an irreplaceable fundamental to secure the future of work (41), extension of OSH management systems has admittedly remained subdued in Southern Africa. Despite some effort by researchers (3, 36, 42–44) to understand the challenges to OSHMSs implementation, there still remains conspicuous evidence in research that the challenges of OSHMSs implementation and their causal factors are not adequately articulated and contextualized in least and developing countries. Previous studies identified challenges to OSHMSs implementation that were broad, generalized and the studies lacked an indication on the universal applicability of these challenges in all industrial sectors (11).

This scenario creates a research gap which could be responsible for the general low uptake of OSHMSs that characterizes most workplaces especially in developing countries (45, 46). As noted by Baghdadi (4) understanding and addressing contextual challenges is key in the endeavor to promote effective implementation of OSHMSs, improve working conditions and managing other operational facets for improved business performance.

Over the past decade and a half, Zimbabwe has been consistently contributing to the bad global OSH performance as evidenced by its average national Lost Time Injury Frequency Rate (LTIFR) of 2.4 over the same period (47). An extrapolation of this statistic projects a worse situation than revealed considering the unaccounted for statistics from the micro, small to medium enterprises and the informal sector at large, yet most of the working population in Zimbabwe (76%) (48) are estimated to be in the informal sector. Mutetwa (49) is convinced that the Zimbabwe’s bad OSH performance reflects many challenges facing workplaces, chief among them being lack of OSHMSs in most of the highly hazardous industrial sectors. Zimbabwe recorded a pathetic 13% national uptake rate of OSHMSs at workplaces in 2021 (47, 49, 50). Considering the perpetual poor OSH performance that has characterized Zimbabwe for over a decade now and the low uptake of OSHMSs at workplaces, it is regrettable to note that the country is yet to ratify the ILO OSH convention 187 on the Promotional Framework for OSH. Non ratification of Convention 187 by Zimbabwe casts a shadow of doubt on the Government of Zimbabwe’s commitment to establishment of OSHMSs at workplaces.

Mutare is the fourth largest city in Zimbabwe and resembles a valley owing to the conspicuous beautiful mountains surrounding it (51). The economic climate of Mutare city is defined by the dominance of the timber-based manufacturing industry that is serviced by forestry estates that are located in various location across Manicaland province of the country. Mutare has over the years been consistently characterized by serious occupational accidents (50). The manufacturing industry of Mutare is known as the highest contributor of occupational injuries in Mutare owing to the dominance of the highly hazardous timber processing factories exacerbated by non-implementation of safety systems (52–54). Due to the prolonged economic challenges in the country, most companies making up the manufacturing industry of Mutare are experiencing an unabated proliferation of unsafe and unhealthy working conditions.

Timber handling jobs are generally classified as dangerous work accounting for a significant number of occupational injuries and diseases occurring at workplaces (55–57). Reinert (58) presumes the unavailability of OSHMSs in risky occupations to be a serious drawback to the endeavor to prevent occupational accidents and diseases. The perpetual safety and health problems in manufacturing industry of Mutare are worrisome to the Government of Zimbabwe and its social partners (Employers and Employees representative bodies) as they have the propensity to curtail the country’s endeavor to attain sustainable development and decent jobs which are intertwined with the strategic milestones for the realization of the envisaged Upper Middle-Income Economy by 2030. World Congress on Safety and Health (59) and Zwetsloot et al. (42) confirm untrustworthiness of sustainable development if it is not embroiled with amelioration of occupational safety and health risks.

National Social Security Authority (NSSA) is charged with a mandate to promote the establishment of OSHMSs at workplaces in Zimbabwe, however little success has been scored in that regard (47). Despite the overwhelming research-based evidence that the benefits of OSHMSs implementation far outweigh the implementation costs (36, 60) it remains a puzzle why most workplaces in Zimbabwe, manufacturing industry of Mutare included, are still not motivated to adopt a systems approach to OSH management. This scenario confronting many workplaces in Zimbabwe, the manufacturing industry included has not been explored in depth in studies conducted to date and therefore presents a strong case for researchers to propagate more research to close the research gaps on the challenges associated with implementation of OSHMSs and their causal factors. As noted by Nordlöf et al. (43), the challenges and causal factors to OSHMSs implementation are many and differ from one organization to another and from one country to another depending on the environmental context. Studies conducted by Gastauer et al. (61) and Abu Aisheh et al. (12) cemented Nordlöf et al. (43) observation by asserting the distinct OSH challenges presented by different workplace environments and recommending the need to devise customized safety measures that are sensitive to addressing the contextual challenges.

A thorough understanding of peculiar OSHMS implementation challenges bedeviling manufacturing industry of Mutare is therefore critical as it allows the application of the Problem Driven Iterative Adaptation (PDIA) philosophy which is handy in instigating the establishment of problem driven solutions that can be adapted to the environmental context of a particular workplace so as to achieve sustained improvements in OSH performance (62). The observation for the need for further research is supported by Seoke and Kamungoma-Dada (63) who recommended the need for further investigation on OSHMSs implementation challenges that SMEs and large enterprises are faced with. It can therefore be deduced that unless the contextual OSHMSs implementation challenges and their causal factors are understood and cured, uptake of OSHMSs at workplaces in Zimbabwe will remain subdued as all promotional strategies will continue to be theory based without addressing the real impediments to uptake of OSHMSs implementation. Development of a problem driven framework to address OSHMSs implementation challenges in the manufacturing industry of Mutare is thus imperative to offset the challenges around the current universal models that are premised on debatable assumption that workplace conditions are similar. This study therefore sought to examine the challenges associated with implementation of OSHMSs in the manufacturing industry of Mutare, assess the causes to the identified OSHMSs implementation challenges and to develop a context based framework for addressing the OSHMS implementation challenges confronting the manufacturing industry of Mutare.

Against ILO’s global OSH strategy for workplaces to establish OSHMS (64), the study is of global significance as it will generate relevant information about the challenges impeding implementation of OSHMSs in manufacturing industry of Mutare and their causal factors that can effectively be interrogated to establish problem driven solutions aimed at ensuring increased uptake of OSHMSs at workplaces. Improved uptake of OSHMSs is particularly important in bringing about sustainability in the management of current and future occupational safety and health risks not only in manufacturing industry of Mutare but also in other workplaces globally that resemble the Zimbabwe’s political, social and economic environmental context. Furthermore this study is of value in promoting decent work agenda at workplaces which is a critical ingredient for the attainment of the developmental milestones envisaged in Zimbabwe’s National Development Strategy (2021–2025) (NDS1) particularly on the following NDS1 priorities: Health and Well-being, Social Protection, Environmental Protection and Economic Growth and Stability. Ultimately, this study provides solutions to prevention of occupational accidents that can be applied by workplaces not only in least and developing countries but globally in the quest to attain sustainable development goals particularly Sustainable Development Goal (SDG) 3, Target 3.9 on substantially reducing the number of deaths and illnesses from hazardous chemicals, air, water, and soil pollution and contamination and SDG 8, Target 8.8 which advocates for protection of labor rights and promotion of safe and secure working environments for all workers by 2030. This study furthermore presented a demand for the Government of Zimbabwe to bolster the application of Education 5.0 in bringing about contextualized OSH innovation and technological advancement in solving challenges associated with implementation of OSHMSs in manufacturing industry of Mutare thereby providing opportunities for attainment of many positive ripple effects of economic growth in many sectors of the economy by boosting job creation, skills development, business productivity and profitability. Perez -Cerrolaza et al. (27) proved the ability of OSH technology in creating a positive safety culture that significantly reduces the rates of safety incidents in the workplace.

## 2 Materials and methods

### 2.1 Research design

Cortina et al. (65) defines a research design as a systematic procedure for data extraction, analysis, interpretation and reporting in research studies. According to Bryman (66), a deeper understanding of the ontological and epistemological assumptions is critical in aiding the research design. Relativism ontology that projects the subjectivity of the reality was found appropriate for this study taking into cognizant that the perception on the reality about the OSHMSs implementation challenges and their causal factors in the manufacturing industry of Mutare is subjective from one company to another. Empiricism and rationalism (67, 68) were the epistemological assumptions adopted to gain knowledge about OSHMSs implementation challenges confronting the manufacturing industry of Mutare. The ascertained ontological and epistemological assumptions informed the methodological design of a descriptive cross sectional study utilizing a mixed method approach. According to Turner (69), mixed method approach is handy in aiding the extraction of divergent multiple viewpoints, opinions, perspectives and standpoints on the challenges associated with the implementation of OSHMSs in manufacturing industry of Mutare and their causal factors thereby enabling data triangulation which is critical in enhancing the validity of the research findings.

### 2.2 Target population

Banerjee et al. (70) explains population as elements, individuals or units that meet the selection criteria for a group to be studied and from which a representative sample is taken for detailed examination. The study population comprised 30 (100%) manufacturing factories making up the manufacturing industry of Mutare, registered by NSSA with a total employee compliment of 1,356 and seven key OSH stakeholders that included Ministry of Public Service, Labor and Social Welfare (MPSLSW), National Social Security Authority (NSSA), Standards Association of Zimbabwe (SAZ), Academia (Mutare Polytechnical College), International Labor Organization (ILO), Zimbabwe Congress of Trade union (ZCTU) and Employers’ Confederation of Zimbabwe (EMCOZ). The selection of the manufacturing industry of Mutare was chiefly motivated by its known hazardous trait (47) owing to the dominance of the hazardous timber-based factories hence implementation of OSHMSs in the manufacturing industry of Mutare was paramount to prevent the occurrence of occupational accidents and diseases.

The justification for the selection of the identified 7 key OSH stakeholders in the implementation of OSHMSs in the manufacturing industry in Mutare is tabulated in Table 1.

**Table 1:** Key OSH stakeholders and justification for their selection.

Number	OSH stakeholder	Role in the study	Justification for selection
1	NSSA	Interview respondent	NSSA is the competent authority to OSH management in Zimbabwe hence have the necessary expertise to increase implementation of OSHMSs at workplaces
2	MPSLSW	Interview respondent	Ministry of Public Service, Labor and Social Welfare plays a role in enforcing labor standards at workplaces hence they are privy to OSHMSs implementation challenges confronting most workplaces
3	ILO	Interview respondent	ILO is the international body with global technical expertise in OSH management hence their inclusion was handy in providing technical advice for improved implementation of OSHMSs at workplaces
4	Academia	Interview respondent	The academia is involved in conducting research in many disciplines hence its inclusion was important in extracting research backed knowledge on OSHMSs implementation challenges in manufacturing industry of Mutare and in proffering solutions in addressing the challenges
5	SAZ	Interview respondent	SAZ is the statutory body that has the legal mandate to develop various standards and also promote their effective implementation at workplaces hence it will provide information on what needs to be done to ensure adoption of OSHMSs standards in the manufacturing industry of Mutare
6	EMCOZ	Interview respondent	EMCOZ represents employers in Zimbabwe hence it will provide employers perspective on the OSHMSs implementation challenges confronting the manufacturing industry of Mutare.
7	MCTU	Interview respondent	MCTU represents employees in Zimbabwe hence it will provide employees perspective on successful implementation of OSHMSs

### 2.3 Sampling methods, determination of sample size and data collection

A sample size of 312 workers which represented 23% of the study population was determined by applying the Slovin’s formula at 95% confidence level basing on Ellen (71) affirmation of the ability of the Slovin’s formula in sampling a population with a desired degree of accuracy.


$$n=\frac{N}{1+N\;{\left(e\right)}^{2}}$$

Where;

n: sample size.

N: proportion of the population.

e: margin of error = 0.05.

The proportion of the sample size was determined in all the 30 manufacturing factories of Mutare according to the formula (72):


$$n_{1}=\frac{\mathrm{Xn}*312}{N}$$

Where: n_1_ = Proportion of sample size in a particular manufacturing factory.

X_n_ = Number of the workers in a particular manufacturing factory.

N = Total target population in the manufacturing industries of Mutare.

Random sampling technique was deployed to sample the 312 workers for questionnaire administration to ensure equality on the probability of each worker being selected as a questionnaire respondent and also to enable the application of inferential statistics in answering specific research objectives (73, 74). The simple random sample was determined by assigning unrelated sequential numerical values to each worker in all the manufacturing factories making up the manufacturing industry of Mutare, then randomly selecting those values using the lottery method. A self-administered structured questionnaire was utilized to randomly collect data from the sample of 312 workers. A questionnaire was also purposely administered to both company appointed Occupational Safety and Health (OSH) practitioner and a top manager or just the top manager alone in the absence of an OSH practitioner in all the 30 factories making up the manufacturing industry of Mutare to enable elucidation of comparable data. Questionnaire respondents were 30 and 11 for top manager and OSH practitioner, respectively. The rationale behind the selection of workers, OSH practitioner and Top manager is tabulated in Table 2.

**Table 2:** Justification for the selection of workers, OSH practitioner and Top manager respondents.

Respondent	Role in the study	Institutional affiliation	Justification for selection
Worker	Questionnaire and Interview respondent	Company under study	Workers involvement and participation was necessitated by the need to unlock workers perceptions about the real challenges impeding implementation of OSH management systems, their causal factors as well as tapping workers’ opinions on solutions that can be implemented to avert OSHMSs implementation challenges
Top manager	Questionnaire and interview respondent	Company under study	Top managers drive the commitment needed for successful implementation of OSHMSs hence their participation helped in determining their perceptions, views and commitment in solving OSHMSs implementation challenges and their causal factors in Mutare manufacturing industry
OSH practitioner	Questionnaire and interview respondent	Company under study	An OSH practitioner is a competent person responsible for coordinating the implementation of OSHMSs hence have a better appreciation of the importance of a systems approach to OSH management thereby making them well positioned to articulate the challenges associated with OSH management systems implementation in the manufacturing industry of Mutare

Follow up interviews earmarked to collect in-depth information from the workers, safety and health practitioners and top managers’ categories were conducted targeting 10% of interviewee respondents from each category. A total of 31 workers were selected for interview administration. Seven interview respondents were selected from each of the two categories of safety and health practitioners and top managers.

The NSSA factories classification system stratifies factories according to the number of employees as follow; factories with employees in the range of 1–50, 51–99, and 100 and above being classified as C (small), B (medium), and A (large) respectively. Stratified random sampling technique was used to select worker interviewees and stratified purposive sampling technique was applied to select both safety and health practitioner and top manager interviewees based on the strata reflected by the NSSA factories classification system. Purposive sampling technique was employed to select one informant each from 7 key OSH stakeholders (Table 1) for further in-depth interviews. A stratified random sampling technique was deployed to proportionally select 15 (50%) of the 30 factories from each of the categories making up the manufacturing industry of Mutare to extract further data through observations. The sample size for observations was credible enough to guarantee the validity of the research results as it surpassed the threshold of 30% sample size as advocated for by Neuman (75). A predetermined observation checklist was utilized to ensure comprehensive extraction of data during observations that answered the research objectives. Notes and appropriate photographs taken during observations that captured the impact of OSH implementation challenges in the manufacturing industry of Mutare were utilized to augment other data sources.

Pre-testing of all data collecting instruments was done prior to their administration on 30% of respondents from each of the categories (workers, OSH practitioners, top managers and key OSH stakeholders) considering the recognition of the validity of 30% sample size in research (75, 76). The reliability and validity of the questionnaires was further assessed through the application of Cronbach’s Alpha. The cronbach alpha reliability coefficient of 0.85 was achieved and considered acceptable in light of a Taherdoost (74) assertion of the acceptability of a reliability coefficient of 0.70 and above. Secondary data was extracted from several sources that included among others OSH journals, targeted companies’ OSH records and documents, past OSH research papers, ILO OSH conventions, OSH international standards, OSH policy and legislation, NSSA’s records on factory inspection and internet-based OSH databases among other sources. A formal letter that provided a guarantee for confidentiality of data was used to seek unhindered access to both primary and secondary data from participating companies. Written informed consent was sought from all the identified respondents and participating companies to ensure voluntary. Anonymization of research instruments was done to uphold confidentiality in order to dispel fear of victimization that could impact negatively on the response rate. Collection of data was done taking into consideration all the COVID-19 protocols.

Safety culture has been examined in several earlier studies (1, 20, 22, 24–26) that have proved the nexus between existence of safety culture and implementation of OSHMS. Taking cognizance of this revelation, a hypothesis to establish whether there was an association between safety culture and implementation of OSHMSs in the context of the manufacturing industry of Mutare was established as follows:

#### 2.3.1 Hypothesis 1

*H_0_*: There is no association between safety culture and the implementation of OSHMS.

*H_1_*: There is association between safety culture and the implementation of OSHMS.

Thirteen generally agreed factors used to gauge safety culture at a particular workplace (29, 33, 34) were adopted to form the index for measuring safety culture in the manufacturing industry of Mutare. The factors that formed the basis for measuring safety culture were namely; observance of safety rules, reporting unsafe acts and conditions, reporting accidents and incidents, conducting Hazard Identification Risk Assessment (HIRA), employee involvement, peer feedback, safety training, safety communication, utilization of PPE, continuous improvement, safety induction, productivity pressure, and accident investigation. Appropriate questions /statements were formulated for each factor and answered by the respondents using a Likert scale with 5 options; strongly agree, agree, undecided, disagree, strongly disagree. Each of the 13 primary factors that measure safety culture was coded as follows; strongly agree and agree were coded 1(Yes) and undecided, disagree and strongly disagree were coded 0 (No).

Management commitment is critical in driving the implementation of OSHMSs (1, 6, 8, 35–39). Basing on this research backed evidence, a hypothesis to establish whether there was an association between management commitment and implementation of OSHMSs in the context of the manufacturing industry of Mutare was established as follows:

#### 2.3.2 Hypothesis 2

*H_0_*: There is no association between management commitment and the implementation of OSHMS.

*H_1_*: There is association between management commitment and the implementation of OSHMS.

Literature demonstrated that there was no generally established template to measure management commitment. Authoritative sources (35, 36), however presume that management commitment to OSH management can sufficiently be evaluated by considering the following 14 parameters namely: formulation of the SHE policy; visible felt leadership walkabouts; certification of the OSHMS; management participation in safety training; resources for OSH management (OSH Budget); setting of OSH objectives and targets; appointment of an OSH practitioner; OSH appraisal system; participation in risk assessment; participation in safety and health committee meetings; awards/incentive system; safety always the first item on the agenda in all operational meetings; management participation in safety inspections and audits and human resources policies that embrace OSH (induction, on job safety training, formal training, pre, periodic and exit medicals). Questions and statements were designed on the 14 parameters for measuring management commitment to OSHMS and answered using a Likert scale that has 5 options; strongly agree, agree, undecided, disagree, and strongly disagree. Each of the 14 primary factors that measure management commitment was coded as follows; strongly agree and agree were coded as 1(Yes) and undecided, disagree and strongly disagree were coded 0 (No).

### 2.4 Data analysis and presentation procedure

Valid questionnaires were numbered sequentially according to the nature of manufacturing business (T, F, E, and O) and the NSSA factories classification system (A, B, and C). For instance, TA1 was questionnaire number 1 for a respondent in a Timber based manufacturing company that is in class A (has more than 100 workers). A simple excel grid that contained a list of all the numbered respondents and a list of all the questions and the corresponding possible options that were available for the respondents’ choice was prepared to collate data from the questionnaires as shown in Table 3.

**Table 3:** Questionnaire sample findings.

Respondent	Question 1: Gender		Question 2: Age (Yrs)					
	Male	Female	<20	20–30	31–40	41–50	51–60	>60
TA1								
TA2								

Source: Researchers.

The questionnaire data was grouped into defined data sets according to the research objectives and presented in statistical manner using frequent tables to illustrate trends and relationship and then discussed continuously in a descriptive manner.

The independent variables in this study were (safety culture and management commitment) and the dependent variable was ‘implementation of OSHMS’. Possible responses to questions on the questionnaires that contained variables of interest were coded as follows; Yes being represented by 1 and No being represented by 0. Chi Square test of independence was used to test the association between variables of interest. Chi-square tests were performed at 5% significance level taking cognizance of the acceptability of a 5% margin of error in Social Sciences (77, 78). SPPS software version 16 was used to present the results of the Chi square test of independence in the form of cross tabulations showing the observed and expected counts, pearson chi-square test result and Cramer’s V and Phi coefficient results. The *p*-value, or Asymptotic Significance of the Pearson Chi – square test was used determine the existence of a statistically significant relationship between the variables of interest. The p-value that was less than 0.05, resulted in the null hypothesis being rejected and a conclusion being made that there is a statistically significant relationship between the variables. The opposite was true for p- value greater than 0.05. Cramer’s V and Phi coefficient are vital measures for assessing the strength of association between two categorical variables (79). The two measures were applied to assess the strength of association between independent variables (safety culture, management commitment) and dependent variable (implementation of OSHMS) with a normalized value between 0 and 1. The Phi and Cramer’s V results were interpreted in accordance with Table 4.

**Table 4:** Phi and Cramer’s V interpretation.

Phi and cramer’s V	Interpretation
>0.25	Very strong
>0.15	strong
>0.10	moderate
>0.05	weak
>0	No or very weak

Source: Akoglu (105).

The statistical data was interpreted to make inferences about the influence of safety culture and management commitment on implementation of OSHMSs, information of which was described qualitatively to compliment other data sources.

#### 2.4.1 Interview, observations and secondary data analysis and presentation

Data from interviews was extracted from the written notes and recorded audios and organized according to the research objectives. A comparative analysis was done to synthesize and group the data into defined data sets according to similarities and differences that would have been seen in the respondents’ responses. Based on the research objectives, similar thoughts from the interview data were quantified, tabulated and presented in a descriptive manner to augment other data sources. Notes and observation checklists from direct observations were analyzed in line with the research objectives and discussed continuously in a descriptive manner to complement other data sources. Photographs taken during direct observations in the manufacturing industry of Mutare were analyzed and described in a continuous prose in line with the research objectives. Secondary data extracted from identified secondary data sources was synthesized and relevant data was reviewed in a descriptive manner in all the sections of the research.

#### 2.4.2 Application of the problem driven iterative adaptation philosophy

**Figure 1:**
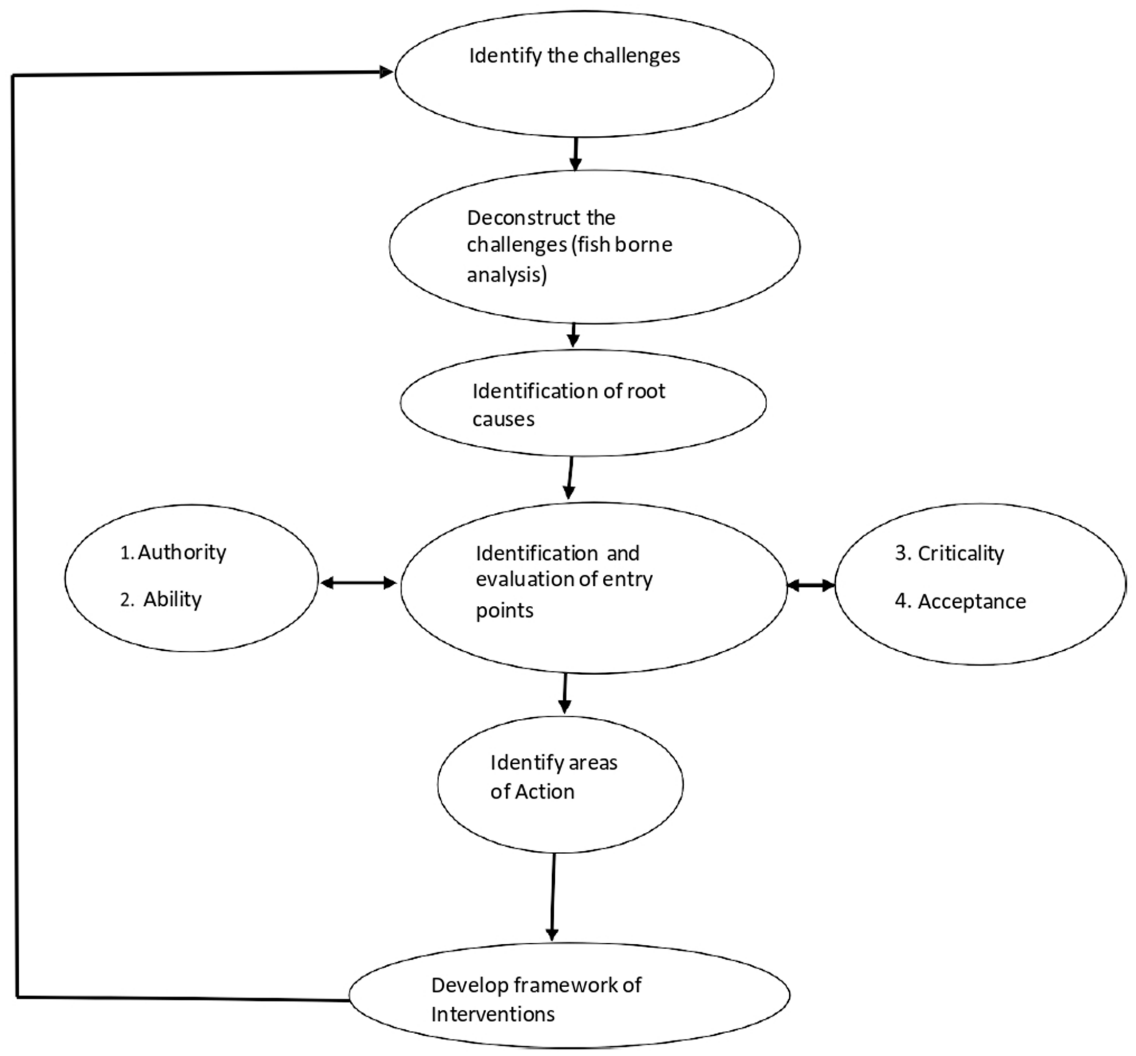
Schematic representation of PDIA strategy. Source: Researchers.

Problem Driven Iterative Adaptation (PDIA) philosophy (Figure 1) (62) was applied to identify root causes to OSHMSs implementation challenges in the manufacturing industry of Mutare. According to Lant et al. (80), the PDIA methodology is a deductive process that is anchored on fish-borne analysis (cause- effect analysis) and that rejects interventions as pure implementation of one size fits all models and good practices from other contexts and instead focuses on problem driven strategies that yield concrete solutions that are in context with the environmental setting rather than solution driven strategies. Evaluation of each identified cause was done in terms of authority, ability, criticality, and acceptance. On a scale of 1–10 the causal factors were ranked to determine entry points of intervention, 1 being low and 10 being high. The PDIA strategy was applied to systematically generate a framework of interventions to OSHMSs implementation challenges in manufacturing industry of Mutare taking into consideration opportunities for experiential learning, coordination, and collaborative approach to counteract the challenges.

A broad-based approach that incorporated input from the Government and its social partners, employers, employees and other key OSH stakeholders’ was used in the development of the framework of interventions to solve the challenges associated with OSHMSs implementation in line with the spirit of inclusivity enunciated by ILO OSH conventions 155 (81) and 187 (82). Soliciting for input from all relevant stakeholders was motivated by Jilcha (83) assertion that effective implementation of OSH programs demands the responsibility of both internal and external stakeholders such as Government, Unions, Insurance agencies, regulatory authorities among others.

### 2.5 Research ethics

Ethical conduct has increasingly received greater attention globally in the field of research in response to society’s expectation of greater accountability (70). Informed consent to participate in the study voluntarily without any form of coercion was sought from both targeted respondents and participating companies. The purpose and significance of the study was fully explained to all the targeted respondents and participating companies to enable them to make an informed decision on whether to participate or not. The consent form among other things included a clause that provided assurance to both the participating companies and respondents on the confidentiality and anonymization of the data sources thereby allaying possible psychological risk of victimization that impacts negatively on the response rate. Due to the sensitivity associated with use of photographs in any research study, the researcher sought informed consent from targeted respondents and participating organizations on the need to take specific photographs during observations in order to compliment other data sources.

## 3 Results

### 3.1 Challenges associated with implementation of OSH management systems in manufacturing industry of Mutare

From a worker perspective, the five major challenges impeding the implementation of OSHMSs were inadequate financial resources to implement and sustain a functional OSHMSs (75.5%), production taking priority ahead of OSH (72.2%), lack of employee participation and involvement (70%), lack of leadership and top management commitment (68%) and lack of qualified and competent OSH practitioners (68%). In sync with the general views of employees, the majority of OSH practitioners were convinced that the topical challenges that have perpetuated the bottleneck of non-implementation of OSHMSs include inadequate financial resources (70%) to counteract the high investment required to implement and sustain the OSHMSs, a culture by the generality of workplaces of viewing safety as a threat to attainment of production targets (70%), lack of leadership and commitment (60%), suboptimal to non-existence OSHMSs implementation knowledge (60%) and lack of national cohesion to promote OSHMSs implementation (60%). The view of the OSH practitioners that inadequate financial resources was a major challenge impeding implementation of OSHMSs resonated well with Shabani et al. (3) study that identified lack of resources among others as a challenge militating against implementation of OSH programs. Interviewees from the Standards Association of Zimbabwe (SAZ) singled out inadequate financial resources owing to a difficult socio-economic environment and lack of qualified and competent OSH practitioners as chief challenges confronting the manufacturing industry of Mutare in general and especially in the micro, small to medium enterprises. NSSA interviewees were agreed that inadequate financial resources, lack of leadership and commitment and lack of safety culture were among the top challenges affecting implementation of OSHMSs by manufacturing industry of Mutare.

Furthermore, NSSA bemoaned unavailability of legislation that advocates for establishment of OSHMSs as a serious impediment to OSHMSs implementation. In line with the thinking of the generality of the respondents, the major employee representative body in Zimbabwe (ZCTU) highlighted lack of leadership and commitment, limited to no knowledge on how to implement OSHMSs, lack of qualified and competent safety and health officers and misconception that safety is a threat to attainment of production targets as top challenges impeding implementation of OSHMSs in the manufacturing industry of Mutare. However, ZCTU was not convinced that lack of adequate financial resources was a major challenge considering that OSHMSs were also conspicuously absent even in organizations that were posting huge profit margins. The major employer representative body (EMCOZ) corroborated with ZCTU’s view by asserting the tendency by most employers of using lack of adequate financial resources as a scapegoat for their lack of leadership and commitment to OSHMSs implementation. ILO representative identified lack of leadership and commitment at both national and enterprise level as one of the major challenges impeding wide spread adoption and implementation of OSHMSs at workplaces. Application of the Chi square test at 5% significance level with 1 degree of freedom revealed a P_value_ of 0.000 which was less than the significance level of 0.05 significance level (as shown in Tables 5, 6). The p- value was less than the 0.05 significance level resulting in the null hypothesis (H_0_) being rejected and the alternative hypothesis (H_1_) being accepted. A conclusion was drawn from the test result that there was an association between management commitment and implementation of OSHMS implementation. Application of Phi and Cramer’s V to assess the strength of association between management commitment and implementation of OSHMS revealed a Phi and Cramer’s V value of 0.62 (Table 7) which was greater than 0.25 thereby implying the existence of a very strong relationship between management commitment and implementation of OSHMSs. This statistical data confirmed the general conviction by respondents that lack of management commitment was a major impediment to uptake of OSHMS in the manufacturing industry of Mutare.

**Table 5:** Management commitment * OSHMS implementation cross tabulation.

Count				
		OSHMS Implementation		Total
		OSHMS not implemented	OSHMS implemented	
Management commitment	Disagree	90	0	90
	Agree	40	50	90
Total		130	50	180

**Table 6:** Pearson chi-square test results for association between management commitment and OSHMS implementation.

	Value	df	Asymp. sig. (2-sided)	Exact sig. (2-sided)	Exact sig. (1-sided)
Pearson chi-square	69.231^a^	1	0.000		
Continuity correction^b^	66.489	1	0.000		
Likelihood ratio	89.050	1	0.000		
Fisher’s exact test				0.000	0.000
Linear-by-linear association	68.846	1	0.000		
N of valid cases^b^	180				

a 0 cells (0.0%) have expected count less than 5. The minimum expected count is 25.00.

b Computed only for a 2×2 table.

**Table 7:** Phi and Cramer’s V symmetric measures for strength between employee involvement and OSHMS implementation.

		Value	Approx. Sig.
Nominal by nominal	Phi	0.620	0.000
	Cramer’s V	0.620	0.000
N of Valid Cases		180	

**Figure 2:**
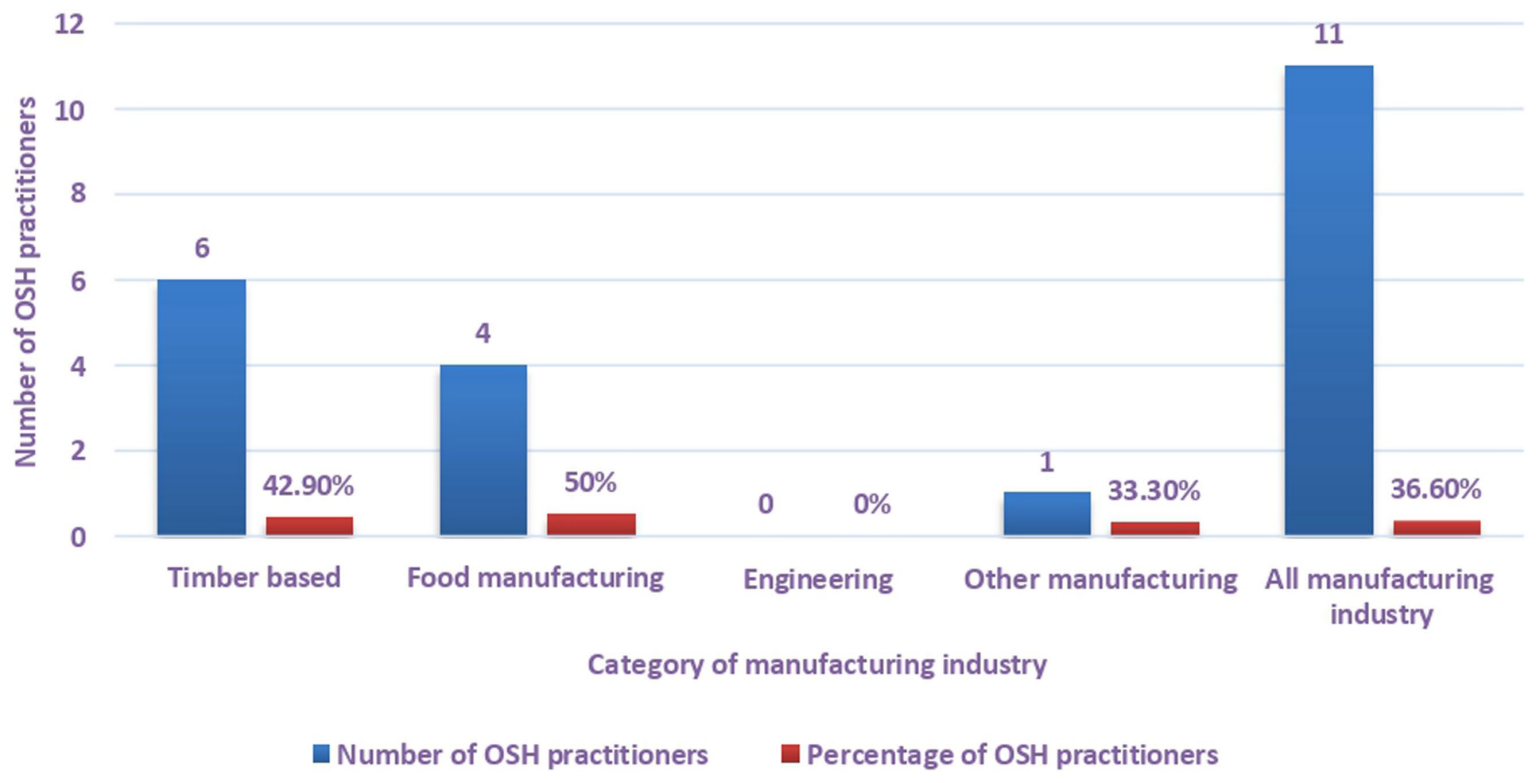
Distribution of OSH practitioners in manufacturing industry of Mutare.

A closer analysis from the results reveals that OSH practitioners were only evident in 36.6% of the workplaces making up the manufacturing industry of Mutare as depicted in Figure 2.

**Figure 3:**
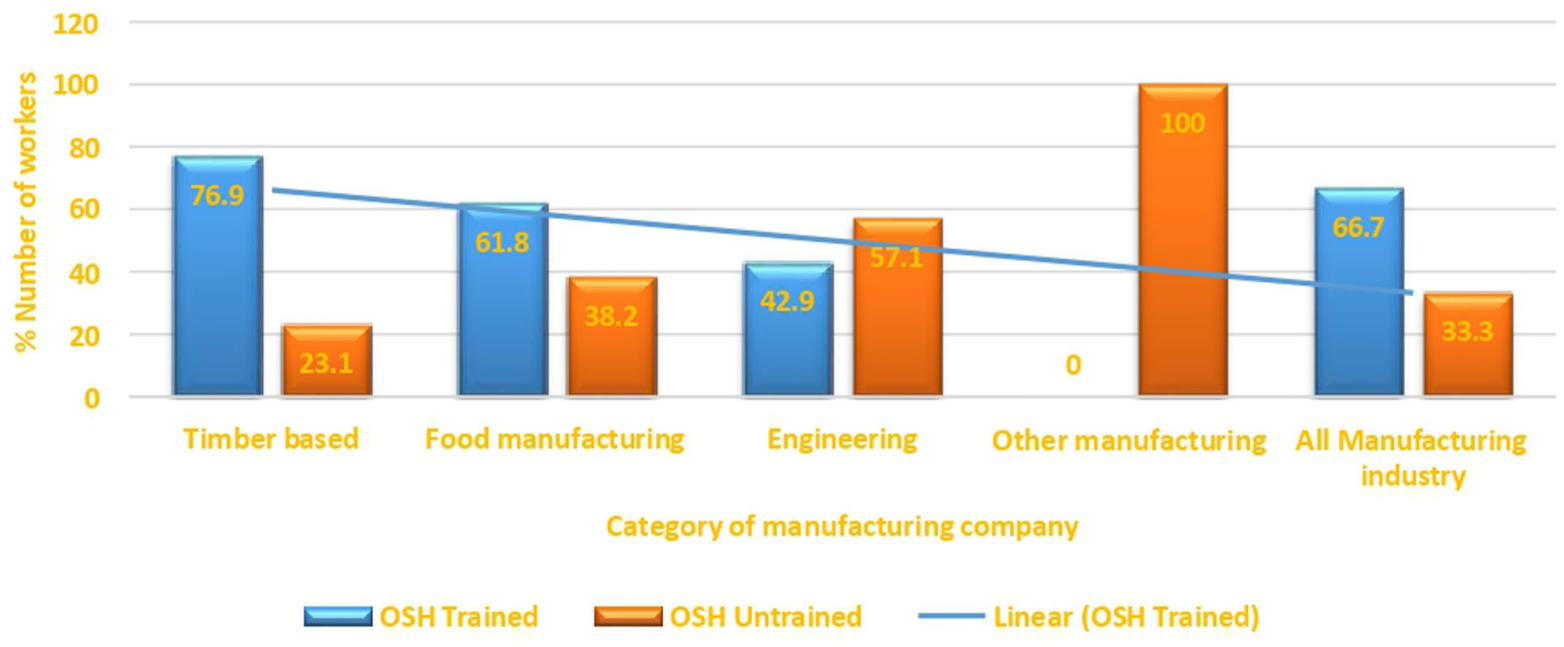
OSH training of workers in manufacturing industry of Mutare.

A categorical analysis suggests that most of the OSH practitioners were found in food (50%) and timber-based manufacturing companies (42.9%). Results show that OSH practitioners were conspicuously absent in engineering companies. A further analysis of all the OSH practitioners identified revealed that 80% had formal educational qualification on OSH management or OSH related field while 20% unqualified OSH practitioners only went to school up to high school level. It is apparent from the results (Figure 3) that there is a noticeable linear trend of a decrease in the number of workers who were trained in OSH starting from timber-based manufacturing category that had the greatest number of OSH practitioners (76.9%) to the Other manufacturing category that did not have OSH practitioners (0%).

**PLATE 1:**
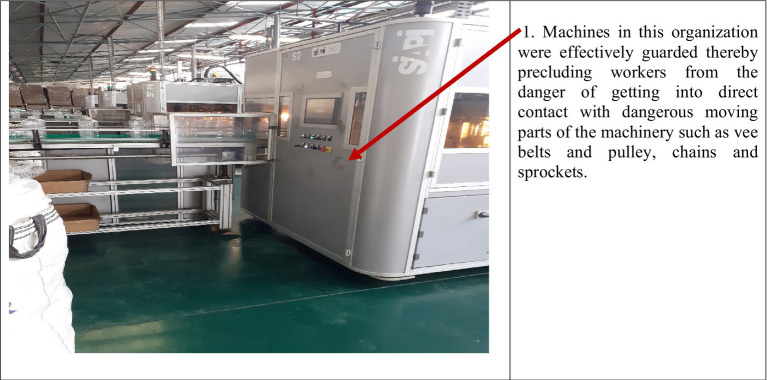
Application of the PDIA strategy. Source: Researchers’.

**PLATE 2:**
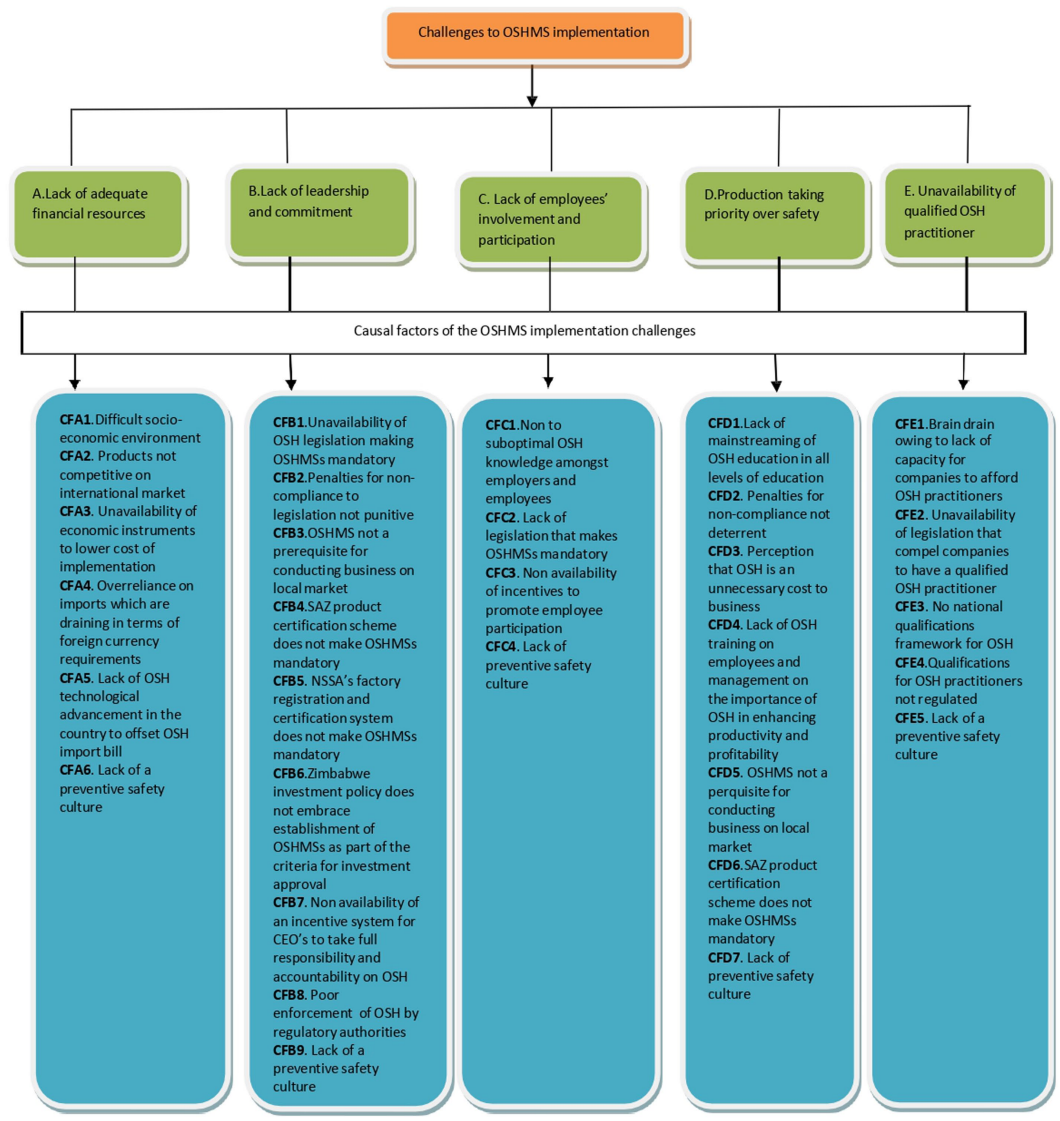
Unsafe working conditions in an organization without a functional OSHMS. Source: Field data (2024).

On a comparative basis, results show that generally organizations that did not implement OSHMSs were characterized by poor safety and health conditions and practices as compared to organizations that had functional OSHMSs. Plate 1 is indicative of poor working conditions that exposed workers to occupational risks in an organization that did not have a functional OSHMS. Conversely Plate 2 is indicative of safe working conditions that were evident in an organization with functional OSHMS. Further analysis of secondary data revealed higher incidences of accidents in organizations without functional OSHMS than in those with functional OSHMSs.

### 3.2 Causes of the challenges to implementation of OSHMSs in the manufacturing industry of Mutare

Most of the workers were convinced that the top three causes of the challenges to non-implementation of OSHMSs in the manufacturing industry of Mutare were unavailability of incentives to lower the cost of implementation (75.5%), unavailability of punitive penalties for non-compliance to OSH legal requirements (75%) and off the shelf OSH management systems imposed without modification to suit organizational context. A significant number of workers also cited inadequate to no training of employees on OSH (68.3%), unavailability of a legal framework to compel OSHMS implementation (59.9%) and the non-unavailability of legislation that makes existence of qualified OSH practitioners mandatory (58.8%) as other causes of non-implementation of OSHMSs. There was convergence of views from key OSH stakeholders that the top five causal factors to OSHMSs challenges were: Lack of management and employee training on the importance of OSHMSs (100%), unavailability of punitive penalties for non-compliance to OSH legal requirements (93.8%), unavailability of incentives to lower costs associated with OSHMSs implementation (93.8%), off the shelf OSHMSs imposed without modification to suit organizational context (87.6%). The academia interviewee was convinced that wide spread unavailability of qualified and competent OSH practitioners in most manufacturing companies was a major causal factor that triggers the manifestation of several operational shortcomings in the implementation of OSHMSs.

Safety and health practitioners’ opinions on the causal factors was not a migration from workers and key OSH stakeholders views as they had consensus that non availability of legislative framework to compel OSHMS implementation (60%), unavailability of incentives to lower OSHMSs implementation costs (60%) and unavailability of legislation that makes existence of qualified OSH practitioners mandatory (59%) were the chief culprits exacerbating OSHMSs implementation challenges in the manufacturing industry of Mutare. The generality of the top managers were convinced that the bottleneck of having off the shelf OSHMSs that are too complex to implement especially for smaller workplaces was an important causal factor contributing to the reluctance in implementation of OSHMS exhibited by most factories making up the manufacturing industry of Mutare. NSSA, a competent authority in the administration of OSH in the country regarded lack of a preventive safety culture as a cross cutting causal factor (CF) to the manifestation of the generality of the challenges to OSHMSs implementation. In tandem with the authoritative assertion by NSSA, application of Chi square test at 5% significance level with 1 degree of freedom revealed a P_value_ of 0.000 which was less than the 0.05 significance level (as shown in Tables 8, 9). The p- value was less than the 0.05 significance level resulting in the null hypothesis (H_0_) being rejected and the alternative hypothesis (H_1_) being accepted. A conclusion was drawn from the test result that there was an association between safety culture and implementation of OSHMS implementation. Application of Phi and Cramer’s V to assess the strength of association between safety culture and implementation of OSHMS revealed a Phi and Cramer’s V value of 0.96 (Table 10) which was greater than 0.25 thereby implying the existence of a very strong relationship between safety culture and implementation of OSHMSs.

**Table 8:** Safety culture * OSHMS implementation crosstabulation.

Count				
		OSHMS implementation		Total
		OSHMS not implemented	OSHMS implemented	
Safety culture	Disagree	127	0	127
	Agree	3	50	53
Total		130	50	180

**Table 9:** Pearson chi-square test results for association between safety culture and OSHMS implementation.

	Value	df	Asymp. sig. (2-sided)	Exact sig. (2-sided)	Exact sig. (1-sided)
Pearson chi-square	1.659E2^a^	1	0.000		
Continuity correction^b^	161.223	1	0.000		
Likelihood ratio	189.646	1	0.000		
Fisher’s exact test				0.000	0.000
Linear-by-linear association	164.971	1	0.000		
N of valid cases^b^	180				

a 0 cells (0.0%) have expected count less than 5. The minimum expected count is 14.72.

b Computed only for a 2×2 table.

**Table 10:** Phi and Cramer’s V symmetric measures for strength between employee involvement and OSHMS implementation.

		Value	Approx. sig.
Nominal by nominal	Phi	0.960	0.000
	Cramer’s V	0.960	0.000
N of valid cases		180	

**Figure 4:**
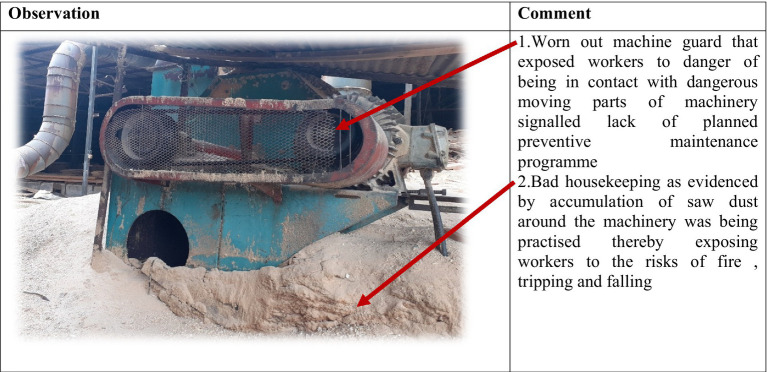
Good safety conditions and practices in an organization with functional OSHMS. Source: Field data (2024).

Systematic application of the PDIA strategy to the identified challenges of OSHMSs implementation revealed that the causes to the challenges are multifactorial as depicted in Figure 4.

Figure 4 demonstrates that each of the challenges affecting implementation of OSHMSs is influenced by several causal factors hence it is imperative that a causal factor-based approach be adopted and implemented at both national and enterprise levels to ameliorate the challenges. Lack of a safety preventive culture emerged as a cross cutting causal factor affecting all the identified OSH implementation challenges pointing to the importance of having interventions at both national and enterprise levels earmarked to promote the creation of a safety preventive culture.

An evaluation of the challenges to OSHMSs implementation using the PDIA evaluation matrix revealed the following entry points of intervention as depicted in Table 11.

**Table 11:** PDIA evaluation matrix for determination of entry points to causal factors for OSHMSs implementation challenges.

Causal factor	Evaluation						
	Authority	Ability	Acceptance	Criticality	Total point	Total point as %	Entry points
Challenge A: Lack of adequate resources							
CFA1	1	1	1	9	12	30	
CFA2	7	7	6	8	28	70	*
CFA3	6	7	8	8	29	72.5	*
CFA4	1	2	7	8	19	47.5	
CFA5	7	8	9	10	34	85	*
CFA6	8	8	9	10	35	87.5	*
Challenge B: lack of leadership and commitment							
CFB1	5	5	8	10	28	70	*
CFB2	5	2	5	8	20	50	*
CFB3	2	1	6	8	17	42.5	
CFB4	2	2	7	9	20	50	*
CFB5	2	6	8	10	26	65	*
CFB6	1	1	5	9	16	40	*
CFB7	7	8	8	9	32	80	
CFB8	7	9	9	10	35	87.5	*
CFB9	8	8	9	10	35	87.5	*
Challenge C: lack of employee involvement and participation							
CFC1	8	9	10	10	37	92.5	*
CFC2	5	4	7	10	26	65	*
CFC3	9	9	9	10	37	92.5	*
CFC4	8	8	9	10	35	87.5	*
Challenge D: production taking priority over safety							
CFD1	5	7	7	8	27	67.5	*
CFD2	5	2	5	8	20	50	*
CFD3	8	8	8	9	33	82.5	*
CFD4	8	9	9	10	36	90	*
CFD5	2	1	6	8	17	42.5	
CFD6	2	2	7	9	20	50	*
CFD7	8	8	9	10	35	87.5	*
Challenge E: unavailability of qualified OSH practitioner							
CFE1	1	1	6	9	17	42.5	
CFE2	3	3	8	10	24	60	*
CFE3	3	3	7	9	22	55	*
CFE4	3	3	8	9	23	57.5	*
CFE5	8	8	9	10	35	87.5	*

NB: CFA, Causal factor A; CFB, Causal factor B; CFC, Causal factor C; CFD, Causal factor D; CFE, Causal factor E. *Denotes entry points of interventions.

There are multiple entry points to addressing each of the challenges of OSHMSs implementation as reflected by the PDIA evaluation matrix (Table 11). A conclusion can be drawn that there is a need to adopt a multiple interventions approach that entail implementing diverse interventions simultaneously to buffer the impact of the challenges thereby ultimately contributing to improved implementation of OSHMSs.

### 3.3 Suggested framework for solving OSH implementation challenges in Mutare manufacturing industry

Based on the identified entry points (Table 2), a deductive approach was used in suggesting the following framework of interventions for solving the challenges associated with OSHMSs implementation as shown in Table 12. It is evident from the framework of interventions that a multi-stakeholder approach is needed to address the challenges to OSHMSs implementation in the manufacturing industry of Mutare.

**Table 12:** Framework for solving OSHMSs implementation challenges in manufacturing industry of Mutare.

OSHMS implementation challenge	Entry point	Mitigatory measure/s
A. Lack of adequate resources	CFA2	Encourage adoption of standards. SAZ must come up with standards promotional strategies targeting all companies making up the manufacturing industry of Mutare. More so SAZ must offer technical assistance to companies at affordable rates to ensure widespread application of standards that give a competitive advantage on the international markets
	CFA3	1. Government should provide tax exemption on importing OSH technology and material to help create more resources for companies to counter budgetary constraints on OSHMSs implementation and sustenance 2. NSSA should offer reduced workers compensation and insurance fund premiums for good OSH performance as an incentive to create the appetite for companies to implement OSH management systems
	CFA5	1. Government should promote OSH research at both national (through the research council of Zimbabwe (RCZ) and institutions of higher education) and enterprise level by creating an OSH research fund to offer grants for targeted OSH research meant to solve OSH problems through technological advancement in line with Article 4; 3[e] of ILO Convention 187 (70) which advocates for a national OSH system that include research on occupational safety and health 2. Government and research institutions such as SIRDC should support the establishment of innovation hubs focussing on OSH so as to ensure most OSH materials are manufactured and supplied locally to ease the aggregate demand of foreign currency on manufacturing companies.
	CFA6	1. In line with R 197 of ILO Convention 187 part 11(5) (106), NSSA, as the competent authority in OSH must support the creation of a preventive safety culture by intensifying delivery of OSH education and training targeting company executives, top management, supervisors, workers and their representatives 2. NSSA should collaborate with Ministry of Higher and Tertiary Education, Innovation Science and Technology Development to introduce occupational safety and health concepts and competencies, in educational and vocational training programs so as to build a national preventive OSH culture
B. Lack of leadership and commitment	CFB1	Government and its social partners (most employer representative and employee representative bodies) should reform the OSH legislation to ensure that it incorporates basic elements of an OSHMS as mandatory requirements
	CFB2	Ministry of Public, Service, Labor and Social Welfare (MPSLW) should collaborate with NSSA to periodically review the OSH laws with a view to ensure penalties for non-compliance to OSH requirements are deterrent in line with Article 4; 2c of ILO Convention 187 (106) that require a national OSH system to include mechanisms for ensuring compliance with national laws and regulations.
	CFB4	SAZ should revolutionize its product certification system by making basic elements of OSHMSs mandatory in line with current global trends that places a human being as central to sustainable development
	CFB5	NSSA should review conditions for factory registration under the Factories and Works Act Chapter 14:08 by incorporating a requirement for basic elements of an OSHMS to be mandatory
	CFB6	Government should review the investment policy to including a requirement for investor to comply with OSH laws as well as basic elements of OSHMS
	CFB8	1. NSSA should mobilize adequate resources to ensure availability of adequate technical, material, and human resources for OSH enforcement 2. NSSA should provide technical assistance to workplaces to promote self-compliance as an ethical conduct and duty of care on the part of organization 3. ILO should periodically offer technical assistance to OSH inspectors to sharpen their skills and technical knowhow on how to execute an effective inspection
	CFB9	1. NSSA should invest in providing OSH education and training targeting company board members, executives and top managers so as to stimulate strong leadership and commitment in OSH by creating sense of responsibility and accountability 2. Government and its social partners should devise an OSH incentive system targeting company executives that recognizes good safety performance thereby creating a healthy competition that promotes OSH leadership and commitment at the highest level 3. NSSA should collaborate with Ministry of Higher and Tertiary Education, Innovation Science and Technology Development in promoting and supporting the incorporation of OSH modules in all programs offered by higher educational institutions
C. Lack of employee involvement and participation	CFC1	NSSA should roll out on a periodic basis some tailor-made OSH training and awareness programs targeting employees in the manufacturing industry of Mutare in line with Vision Zero concept on ‘invest in people motivate by participation’.
	CFC2	Government should review the OSH laws to ensure that they incorporate the need for workplaces to have functional OSH management systems for continual improvement
	CFC3	NSSA should concertize organizations to establish OSH incentives at enterprise level that help to boost employees’ interest thereby promoting their involvement and participation
	CFC4	1. Organizations must invest in education and training of workers on an ongoing basis 2. Organizations must promote mutual learning and networking among employees by coming up with OSH competitions and joint OSH inspections or audits in line with the Vision Zero concept’s value of mutual learning and networking 3. NSSA should collaborate with ZCTU in coming up with national, regional and enterprise level OSH awareness campaigns to boost employee interest in OSH management
D. Production taking priority over safety	CDF1	Ministry of higher and tertiary education should collaborate with NSSA in mainstreaming OSH in all academic programs offered at higher and tertiary educational institutions in line with R 197 of ILO Convention 187 part 11(5) (106) that advocates for introduction of occupational safety and health concepts and, where appropriate, competencies, in educational and vocational training programs
	CDF2	Ministry of Public, Service, Labor, and Social Welfare (MPSLW) should collaborate with NSSA to review the OSH laws with a view to strengthen penalties for non-compliance to OSH requirements in line with Article 4; 2c of ILO Convention 187 (70) that requires a national OSH system to include mechanisms for ensuring compliance with national laws and regulations.
	CDF3	1. In line with Article 14 of ILO Convention 155 (ILO 1981), Ministry of Higher and Tertiary Education, Innovation Science and Technology Development should collaborate with NSSA in coming up with measures to include questions of occupational safety and health and the working environment at all levels of education and training to as to build an OSH preventive culture in graduates that later assume leading roles in industry 2. NSSA should roll out training programs periodically targeting top management and executives to help boost OSH awareness among the decision makers
	CDF4	In line Article 19 (d) of C 155 (81), NSSA should devise and roll out OSH training program targeting top management, executives and employees to project the importance of OSH so as to achieve adequate levels of OSH management awareness
	CDF6	SAZ should revolutionize its product certification system by making basic elements of OSHMSs mandatory in line with current global trends that places humans as central to sustainable development
	CDF7	1. NSSA should invest in providing OSH education and training targeting top managers, executives and company board members to bolster their leadership and commitment in OSH management 2. Government and its social partners should devise an OSH incentive system for company executives that recognize good safety performance thereby creating a healthy competition that promotes OSH leadership and commitment at the highest level 3. NSSA should collaborate with Ministry of Higher and Tertiary Education, Innovation Science and Technology Development in promoting and supporting the incorporation of OSH modules in all programs offered at higher and tertiary educational institutions 4. Government should expedite the review of the OSH law to ensure that it incorporates the need for workplaces to have functional OSHMSs for continual improvement
E. Unavailability of qualified OSH practitioners	CFE2	Ministry of Public Service, Labor and Social Welfare should collaborate with NSSA in reviewing OSH legislation to ensure the incorporation of a requirement for workplaces to have a qualified OSH practitioner
	CFE3	In line with Article 5(c) of C 155 (81) which demands qualifications of persons involved in OSH administration to be defined, MPSLSW should collaborate with NSSA in consultation with the Zimbabwe Institute of OSH (ZIOSH) in reviewing the National OSH policy to ensure it provides a national framework for OSH qualifications
	CFE4	1. In line with Article 5(c) of C 155 (81) which demands qualifications of persons involved in OSH administration to be defined, MPSLSW should collaborate with NSSA in consultation with Zimbabwe Institute of OSH (ZIOSH) in reviewing OSH legislation to incorporate the minimum basic qualifications for OSH practitioners 2. MPSLSW and NSSA should review OSH legislation to compel companies to employ OSH practitioners accredited by ZIOSH or those who would have undergone professional OSH course such as Occupational Safety, Health and Environmental Management Course (OSHEMAC) or any other recognized by NSSA as sufficient to ensure effective delivery of OSH
	CFE5	1. NSSA should invest in providing OSH education and training targeting top managers, executives and company board members to bolster leadership and commitment in OSH management at the highest organizational level. 2. Government and its social partners should devise an OSH incentive system for company executives that recognize good safety performance thereby creating a healthy competition that promotes OSH leadership and commitment at the highest level 3. NSSA should work with Ministry of Higher and Tertiary Education, Innovation Science and Technology Development in promoting and supporting the incorporation of OSH modules in all programs offered at higher and tertiary educational institutions

## 4 Discussion of the results

### 4.1 Challenges associated with implementation of OSH management systems in manufacturing industry of Mutare

The most prominent challenges associated with implementation of OSHMSs in manufacturing industry of Mutare were lack of adequate resources, lack of leadership and commitment, lack of employee involvement and participation, production taking priority ahead of safety and lack of qualified OSH practitioners. According to Wang et al. (84), lack of adequate resources is a serious impediment to OSHMSs implementation as it implies lack of capacity to offset the huge initial cost associated with OSHMSs implementation and the perpetual costs of sustaining the system. Furthermore, it must be noted that lack of adequate resources is generally a problem more pronounced in micro, small and medium enterprises (MSMEs) than in large enterprises, hence the noticeable disparities in uptake of OSHMSs as large enterprises tend to afford the technical, material and human resources required to effectively implement OSHMS owing to their large resource base. Without adequate resources an organization will be handicapped in many areas that include provision of PPE, plant and equipment maintenance and general compliance with OSH legislation requirements. Conversely, the challenge of inadequate resources can be a direct result of another challenge of lack of leadership and commitment. Generally, the challenge of lack of resources is evident in workplaces that lack leadership and commitment hence it is normally used as a scapegoat for non-implementation of OSHMSs. This reasoning is cemented by ISSA’s (36) firm view that the extent to which resources are availed for OSH is a barometer to measure the level of OSH leadership and commitment in an organization. This line of thinking by ISSA (36), can be a solid reason why OSHMSs can be conspicuously absent even in organization that have a large resource base thereby affirming the importance of leadership and commitment in unlocking resources for OSH administration. Chi square test applied at 5% significance level revealed the existence of an association between management commitment and implementation of OSHMSs. The Phi and Cramer’s V value confirmed a very strong association between management commitment and implementation of OSHMSs. These statistical results solidify the golden rule number one on ‘Leadership and commitment’ of the Vision Zero concept that asserts sound leadership and commitment as the driving force behind successful implementation of safety systems. As noted by ISSA (36), the quality of leadership and commitment is a determinant on how attractive, successful, and sustainable the OSHMS will be at a workplace.

Another school of thought seems to suggest that the level of commitment to OSH management is influenced by the size of the workplace (85). Zwetsloot et al. (42) noted that large companies tend to benefit from OSH more than Micro, Small to Medium Enterprises (MSME) because they have the necessary size and structure to facilitate effective implementation of OSHMSs. Against a backdrop of a considerable investment demanded in the implementation and sustenance of an OSHMS, companies tend to gravitate toward investments where the return on investment benefits every employee. This universal understanding by most organizations precipitates a situation where large companies with bigger number of employees are more motivated and committed to implement OSHMS driven by a thrust to benefit more people thereby spreading the cost per person much thinner. Notwithstanding the cost associated with OSHMS implementation, international research on the return on investments in OSH prevention spearheaded by ISSA proved the importance of OSH in transforming the economic fortunes of an enterprise by asserting that every dollar invested in OSH generates a potential benefit of more than two dollars in positive economic effects. Evidence on the ground in both developed and developing countries suggests that certified OSHMSs are only more possible and attractive in large worksites owing to the stringent requirements of OSHMSs standards (43, 86), hence irrelevant in MSMEs that do not have a huge workforce to guarantee translation of OSHMS implementation into direct monetary gain for business sustainability. Based on the scientific evidence in literature, it can be concluded that it is an insurmountable task to expect OSHMSs of the same form and structure to be implemented at the same level for both large enterprises and MSMEs. There is therefore a compelling need for proponents of OSHMSs to consider scaling down OSHMSs requirements for MSMEs to accommodate their simple organizational structures as well as their operational limitations that among other things include limited access to technical, material and human resources, development capital and competitive advantage on markets.

The challenge of lack of employee involvement and participation vindicates long held observation of the centrality of employees in the success of an OSHMS. A study by Shabani et al. (3) identified employee involvement as paramount in boosting the level of safety and health awareness about the importance of OSH among workers which is critical in developing an organizational preventive safety culture. According to the Vision Zero concept golden rule 7 on ‘Invest in people-motivate by participation’, motivating employees by involving them in all safety and health matters is an investment that pays off. As noted by ISSA (36), when employees are involved and consulted their willingness to follow safety rules is improved thereby yielding ripple positive effects of a change in safety culture. Research has proved employee involvement to be having a strong link to employee commitment and improved OSH results (87–89). Lack of employee involvement and participation can be negatively influenced by lack of leadership and commitment of top management in addressing safety and health concerns raised by employees. Organizations with sound preventive safety culture tend to experience highest level of top management commitment and employee involvement and participation (25, 72). Organizations must therefore invest in transforming organizational safety culture as a precursor to employee involvement and participation. Investment in safety education and training and rolling out safety promotional programs such as giving safety performance rewards to employees for good safety performance and creating platforms for mutual learning and networking are viable interventions of buttressing employees’ involvement and commitment.

The challenge of production taking priority over safety reflects lack of a preventive safety culture in an organization that ultimately triggers subdued levels of top management commitment to OSH management. Failure by OSH practitioners to project organizational OSH performance in terms of monetary value is a possible causal factor to the challenge of production taking a center stage ahead of safety. Management tends to appreciate OSH more when the consequences of OSH failure are reflected on the company’s balance sheet in monetary terms (85). Substantial losses to organizational profits arising out of OSH failure will trigger a reaction by management to consider OSH as an irreplaceable value embedded in all business processes. The Chi square test, Phi and Cramer’s V value results that revealed the existence of a strong association between safety culture and implementation of OSHMS cements the importance of a preventive safety culture in OSHMSs implementation as it is consistence with other studies (90, 91) that proved beyond any reasonable doubt the dependability of OSHMSs on the availability of a preventive safety culture. Conversely, other studies (92, 93) affirmed the influence of a safety management systems on building a preventive safety culture thereby asserting the existence of a two way causal relationship between OSHMSs implementation and the creation of a preventive safety culture. This revelation places a demand on organizations to invest in recruiting qualified and competent OSH practitioners with the requisite technical OSH skills to effectively transform organizational safety and health culture.

Timber-based manufacturing companies had the highest number of OSH practitioners (54.5%) as compared to other categories of manufacturing companies. This could be explained by the fact that the highest number of big companies in the manufacturing industry of Mutare were timber based and they supply both the local and international market, hence market forces push them to have an OSH management system which according to Fick (85) can only be effectively attained with the assistance of a qualified and competent OSH practitioner. International markets world over are increasingly placing a demand on suppliers to demonstrate as a condition for product’s acceptance on the market, their commitment to guarantee occupational safety and health for their employees’ health (86, 94). The non-availability of OSH practitioners in engineering firms as portrayed by the results is indicative of workplaces in this category that lack the necessary OSH expertise to spearhead the effective implementation of OSHMS. This bottleneck of non-availability of OSH practitioners is compounded by lack of robust policy and legislative framework that compels the appointment of OSH practitioners at all workplaces. The current Zimbabwe National OSH policy of 2021 (95) only places a demand for an OSH practitioner to be appointed in an organization that would have adopted an OSHMS, hence it is imperative to revisit this policy pronunciation with a view to ensure its widespread applicability. Many scholars globally presume the availability of qualified and competent OSH professionals as a catalyst for establishment of effective OSHMSs at workplaces (96).

The fact that most of the qualified OSH practitioners (75%) had attained a first degree in either OSH or OSH related field is a testimony of the commendable strides that the Zimbabwe’s education sector is making in ensuring the wide-spread establishment of OSH academic programs in several tertiary institutions in the country. Many studies conducted globally attest to the importance of mainstreaming OSH in tertiary education in order to create a preventive safety culture (97, 98). Results further revealed that some employees in the manufacturing industry of Mutare were practicing OSH without the requisite qualifications, a situation that militates against the endeavor to effectively implement OSHMSs. Taking cognizance of the OSH practitioner’s role in effective implementation of OSHMSs that is increasingly being recognized globally, there is now a compelling realization of the need to establish minimum education, experiential, and practice standards for those working in the OSH field. According to Hale (96), there is an overdrive in the developed economies to regulate the entry qualifications and competence for OSH professionals and to ensure that OSH professionals are affiliated to recognized professional bodies. On the contrary, there is suboptimal appetite in developing and least developed countries to ensure the regulation of OSH profession though there is consensus on the criticality of OSH professionals in effective management of OSH at workplaces (8). In line with the 2019 ILO centenary declaration for the future of work, there is a need for the Government as a policy maker to consider coming up with initiatives to increase investment in improving capabilities of OSH practitioners as they are central in curtailing the challenges associated with effective implementation of an OSHMSs.

### 4.2 Causes of the challenges to implementation of OSHMSs in the manufacturing industry of Mutare

Synthesis of the input from research participants and application of the PDIA strategy revealed that the causes of the challenges to implementation of OSHMSs in the manufacturing industry of Mutare are largely multifactorial and require a multifactorial approach to intervention. Prominent among the causes were unavailability of incentives to lower the cost of OSHMSs implementation, unavailability of punitive penalties for non-compliance to OSH legal requirements, unavailability of a legal framework to compel OSHMSs implementation, off the shelf OSHMSs s imposed without modification to suit organizational context, the non-unavailability of legislation that makes existence of qualified OSH practitioners mandatory and unavailability of an OSH qualification framework for OSH practitioners. Unavailability of incentives to lower the cost of OSHMSs implementation makes the cost of OSH investment unsustainable for workplaces that are in the MSMEs category as they will not have sufficient financial capacity to implement and sustain an OSHMS leading to the manifestation of a plethora of OSH deficits.

Studies by several authors on small businesses in Australia (53, 60, 99, 100) attest to a barrier of lack of adequate financial resources as contributing factor to most of health and safety challenges associated with small businesses. The causal factors of unavailability of a legal framework to compel OSHMS implementation and unavailability of punitive penalties for non-compliance to OSH legal requirements are exacerbating the enforcement challenges confronting the generality of regulatory institutions in Zimbabwe that have the mandate to administer OSH at workplaces. Penalties that are currently being applied for flouting of OSH laws are outdated and no longer serving their purpose as legal instruments that instill a preventive safety culture owing to bureaucratic processes associated with review of legislation. It is imperative to highlight that due to the challenges shrouding the prescriptive traditional command approach to OSH governance, the new world order in the field of work is increasingly gravitating toward a goal setting philosophy of self-compliance based on the premise that collectively employers and employees are best placed to identify and control workplace hazards and risks (101).

Off the shelf OSHMSs imposed without modification to suit organizational context is another causal factor highlighted as contributing to the manifestation of the challenges associated with implementation of OSHMSs in manufacturing industry of Mutare. Mandowa et al. (1) literature review on implementation of OSHMSs placed a strong demand on the need for customization of off the shelf OSHMSs taking into consideration rapidly evolving field of work arising out of growth in globalization, technological advancement, work intensification, internet of things, robotics and nanotechnology compounded by changing makeup of the workforce, informality, changing work arrangements and socio economic status of an organization (35, 102). According to Frick (102), the strategy for OSH management has significantly shifted from detailed requirements (“what to do”) toward a proactive prevention (“how to get it done”), through streamlined OSHMSs. Walters and Wadsworth (103) highlighted the significance of presenting low cost, affordable solutions to encourage small businesses to establish functional OSHMSs.

Non-availability of legislation that makes existence of qualified OSH practitioners mandatory is compounded by lack of an OSH qualification framework for OSH practitioners in the country. As noted by ILO, there is no international labor standard that reflects member states agreement on the regulation of OSH professionals; however, the International Network of Safety and Health Practitioner Organizations (INSHPO) published the Occupational Health and Safety Professional Capability Framework - A Global Framework for Practice (Framework) which serves as a foundation for developing international standards of practice for those working in OSH (104). It is paramount to note that the degree and level of inclusion of OSH in the national education systems and trainings largely depend on the level of development of these systems and on specialized personnel available; hence it is imperative for countries to embrace a best fit approach for mainstreaming OSH into academics considering differing environmental contexts.

### 4.3 Suggested framework to solving OSH implementation challenges in manufacturing industry of Mutare

It is evident from the framework of interventions that a multi-stakeholder approach is needed to address the challenges to OSHMSs implementation. This revelation from the results resonates well with the ILO (35) assertion that recognizes the need for strategic collaborations and synergies as critical in sound OSH management at both national and enterprise level. The results could therefore be indicative of the need to reform the national OSHMS framework to ensure that it promotes cooperation, collaboration, and active participation of all key players in OSH management at both national and enterprise level in many spheres of OSH management that include among others integrated OSH inspections, OSH research, OSH promotion and training and OSH medical surveillance.

## 5 Theoretical implications

The findings of this study are largely bolstering the existing literature on OSHMSs and more specifically providing a deeper understanding of the peculiar challenges to OSHMSs confronting the developing countries that are contributing toward a noticeable general subdued uptake of OSHMSs in developing countries in spite of the concerted efforts globally to advance a system approach to OSH management as an irreplaceable fundamental to achievement of OSH sustainability. The contribution of this study to the existing literature and body of knowledge is summed up based on the three objectives that are addressed by this study. Firstly by seeking to examine the challenges associated with implementation of OSHMSs in the manufacturing industry of Mutare, this study enriches the body of OSHMSs literature by giving a deeper exploration of the contextual challenges impeding OSHMSs implementation in manufacturing industry of Mutare thereby providing the basis for scholars to rethink the best approaches to encourage uptake of OSHMSs that are sensitive to solving the contextual challenges besieging workplaces. Currently, as noted by Kunodzia et al. (10), there are few studies that have been conducted today to explore the challenges to OSHMSs implementation affecting workplaces in least developed and developing countries. Furthermore Kunodzia et al. (10) established that generally, studies concerning challenges to OHSMSs implementation were limited largely to systematic literature reviews focusing mainly on developed countries whose political, social and economic environmental context is different to that of the least developed and developing countries. Kunodzia et al. (10) observed that generally, studies concerning challenges to OHSMSs implementation were limited to systematic literature reviews and were conducted in developed countries. Mandowa et al. (1) study warned against the global order of one size fit all approach to implementation of OSHMSs without considering the differing environmental challenges as militating against the endeavor to increase uptake of a systems approach to OSH management at all workplaces. The study makes it clear based on the scientific evidence in literature, that it is a mammoth task to expect OSHMSs of the same form and structure to be implemented at the same level for both large enterprises and MSMEs. This revelation contributes to the body of knowledge by bringing to light a new demand for proponents of OSHMSs to consider scaling down OSHMSs requirements for MSMEs to accommodate their simple structures and inherent operational challenges that include among others limited access to technical, material and human resources and competitive advantage on markets.

Secondly, the study provides insights on the underlying causes of the challenges by assess the causes to the identified OSHMSs implementation challenges. Several studies (3, 29, 36, 42, 44) that have been conducted to provide an understanding of the challenges to OSHMSs implementation were bereft of establishing the specific underlying causes to the challenges. This study capitalizes on this shortcoming by seeking to dig deeper into understanding the underlying causes triggering the manifestation of the OSHMSs implementation challenges. Lindholm et al. (11) noted that previous studies identified challenges to OSHMSs implementation that were broad, generalized and their causal factors are not adequately articulated and contextualized in least developed and developing countries. Against an assertion by Pritchett et al. (62) that advocates for implementation of local solutions to local problem, this study therefore contributes to the enrichment of the existing literature by unearthing the causes to the OSHMSs implementation challenges that are remotely addressed in literature thereby providing a deeper understanding of the root causes of the challenges to OSHMSs implementation in the manufacturing industry of Mutare. This information can form the basis for providing solutions to OSHMSs implementation challenges in other workplaces in Africa and beyond that face similar political, socio-economic environmental context to that of Zimbabwe.

Lastly, the novelty of this study is its ability to build on the understanding of the challenges to OSHMSs implementation and their causes that enabled the development of a framework to circumvent the OSHMS implementation challenges. It is interesting to note that the framework to address the identified OSHMS implementation challenges was established through the application of the PDIA strategy, a strategy that systematically demonstrated that each of the challenges affecting implementation of OSH management systems is influenced by several causal factors hence it was imperative that a causal factor-based approach be adopted and implemented at both national and enterprise levels to ameliorate the challenges. This PDIA strategy was handy as it contributed to the body of knowledge by enabling the identification of the entry points to addressing the challenges which was critical in ensuring optimum utilization of resources through a process of prioritization. This understanding is critical in ensuring the application of several interventions at both national and enterprise level and in providing an understanding that collaboration, cooperation, mutual learning and networking as noted by ISSA (36) are paramount in the quest to achieve successful implementation of OSHMSs. The suggested framework also contributed to new knowledge by revealing an OSH qualification framework gap confronting many developing countries thereby placing a demand on ILO to reflect on the need to develop an international labor standard on the regulation of OSH professionals that serves as foundation for developing international and national standards of practice for OSH professionals. Currently there is no agreed international labor standard that reflects member states agreement on the regulation of OSH professionals serve for the Occupational Health and Safety Professional Capability Framework advanced by the International Network of Safety and Health Practitioner Organizations (INSHPO) (104). Ultimately, the OSH qualification framework gap established by the study places a challenge on countries to embrace a best fit approach to strengthen their OSH qualification frameworks considering differing environmental contexts.

## 6 Practical implications

The world of work is rapidly changing and perpetually confronted with a host of dynamic and unprecedented OSH risks which can only be countered by managing OSH in a sustainable manner. In line with this current global thinking, one of the practical implications of this research is to create undoubtable knowledge and awareness for the Government of Zimbabwe and its social partners (Employer and Employees bodies) to take OSH as a business case and the need for OSHMSs implementation as an irreplaceable fundamental value. This understanding therefore informs the basis for the need to ratify and domesticate the provisions of ILO Convention 187 on the promotional framework for OSH which clearly advocates for a systems approach to effective risk management at the workplace.

The study cemented the need for workplaces to embrace a preventive safety culture as a critical driver in the successful implementation of OSHMS taking cognizance of the compelling fact that organizations with sound preventive safety culture tend to experience highest level of top management commitment and employee involvement and participation (25, 72). The study therefore challenges workplaces to invest in transforming organizational safety culture as a precursor to management commitment and employee involvement and participation. Investment in safety education and training and rolling out safety promotional programs such as giving safety performance rewards to management and employees for good safety performance and creating platforms for mutual learning and networking are viable interventions of buttressing management and employees’ involvement and commitment. On the other end other studies (92, 93) affirmed the influence of a safety management systems on building a preventive safety culture thereby asserting the existence of a two way causal relationship between OSHMSs implementation and the creation of a preventive safety culture. This revelation places a demand on organizations to invest in recruiting qualified and competent OSH practitioners with the requisite technical OSH skills to effectively transform organizational safety and health culture which has the propensity of boosting management and employees’ commitment to OSH.

The study revealed the need for scaled down OSHMSs requirements for MSMEs to accommodate their simple structures and inherent operational challenges, hence the study can practically be applied to reform the Zimbabwe national OSH Policy and Legal framework to ensure the incorporation of a requirement for workplaces to implement some defined minimum basic OSHMSs elements irrespective of the size of the workplaces. Furthermore there is need to put a legal provision to ensure that penalties for non-compliance to OSH regulations are constantly updated to ensure their relevance as legal instruments in order to inculcate a preventive safety culture. These policy and legal reforms are handy in aiding OSH enforcement and compliance which is a necessity in successful safety and health management at workplaces.

Against the recognition by Yangho et al. (101) that the prescriptive traditional command approach to OSH governance is increasingly being superseded by the new goal setting philosophy of self-compliance globally, another practical application of this study is the need for the national OSH promotional framework of Zimbabwe to be refined to ensure that it is more focused on promoting implementation of OSHMSs through scaled up training programs on the importance of OSHMSs targeting decision makers such as company executives and management considering that the ultimate responsibility lies with leadership as far as the decision to implement any occupational safety and health management program is concerned (42).

## 7 Conclusion and recommendations

It can be concluded from the study that the main challenges to OSHMSs implementation in the manufacturing industry of Mutare are lack of adequate resources, lack of leadership and commitment, lack of employee involvement and participation, production taking priority over safety, lack of qualified OSH practitioners among others. The nexus revealed between management commitment and OSHMSs implementation as reflected by inferential statistics presents an inescapable demand for the need to refine both national and enterprise OSH promotional strategies to be more targeted toward boosting leadership and commitment to OSH management. The study identifies the root causes to OSHMS implementation challenges to be multifactorial and demanding a cocktail of interventions to solve them. The existence of an association between safety culture and OSHMSs implementation is indicative of the need for NSSA to embark on a multi-stakeholder approach in reforming the national OSH management framework to ensure that it embraces national OSH programs targeted at building a national OSH preventive culture. It can be extrapolated from the study that there is need for the Government of Zimbabwe and its social partners (employer and employee representative bodies) working in collaboration with the association of OSH professionals (ZIOSH) and the academia to establish a well-defined national OSH qualifications framework aimed at strengthening OSH delivery at both national and workplace level. Furthermore, there is need for the NSSA and ZIOSH to collaborate in the establishment of an accreditation system for OSH practitioners practicing at enterprise level as well as those into OSH consultancy. This will be beneficial in averting the proliferation of unqualified and incompetent OSH practitioners whose services are largely detrimental to effective implementation of OSHMSs at workplaces. The Government of Zimbabwe should demonstrate its commitment to systems approach to OSH management by ratifying ILO Convention 187 on the promotional framework for OSH which will serve as a basis for the Government of Zimbabwe to domesticate implementation of OSHMSs through policy and law intervention. The research outcomes demand that there be collaboration, cooperation and synergies among the Government and its social partners and other key OSH stakeholders (such as NSSA, ZIOSH, the academia) in mainstreaming OSH into all levels of education and training in a manner meeting the OSH training needs of all workers.

## 8 Limitations and future research

This research accommodates several limitations some of which are worth pinpointing for future researchers. The first limitation was that the results of the study emanate from data that was extracted using questionnaires and interviews administered to workers from the manufacturing industry of Mutare, which creates a constraint in the generalizability of the results to other manufacturing industries in other cities and towns of the country. OSHMSs are generally considered as applicable to most industries, however as a result of this limitation, the results of this study may not be applicable in generalizing what could be transpiring in other manufacturing industries nationwide owing to the compelling fact that different manufacturing industries might experience and encounter different levels and types of OSHMSs implementation challenges. Future research is thus recommended to expand the scope of the research to ensure that it is able to cover other manufacturing industries located in other towns and cities of the country in order to obtain a more generalizable result. Secondly, this study utilized a descriptive cross sectional survey, hence was only able to project what is transpiring in the manufacturing industry of Mutare as far as the challenges associated with OSHMS implementation are concerned at the particular point in time. This means that the study could not examine the effects of safety culture and management commitment on OSHMS implementation over a longer period of time. In the future, a longitudinal study should be handy to establish whether there is any variation in the behavior of the same set of variables of interest over a longer period. Furthermore, future research into OSHMSs implementation challenges can be strengthened by broadening it to focus on conducting comparative analyses with other industrial sectors and exploring regional disparities and other regional best practices in terms of interventions. This approach can result in a more comprehensive understanding of the OSHMSs implementation challenges in developing countries thereby informing evidence-based strategies for enhancing sustainable implementation of OSHMSs at workplaces. Another limitation is the existence of a possibility of unexplored confounding factors that might influence the relationships observed on the research findings. It is therefore paramount for future research to explore in depth other potential confounding factors that might influence OSHMSs implementation challenges thereby enriching the academic body of knowledge.

## Data Availability

The original contributions presented in the study are included in the article/supplementary material, further inquiries can be directed to the corresponding author.
